# Evaluation of the IR Biotyper for *Klebsiella pneumoniae* typing and its potentials in hospital hygiene management

**DOI:** 10.1111/1751-7915.13709

**Published:** 2020-11-18

**Authors:** Yanyan Hu, Hongwei Zhou, Jiayue Lu, Qiaoling Sun, Congcong Liu, Yu Zeng, Rong Zhang

**Affiliations:** ^1^ Clinical Microbiology Laboratory School of Medicine 2nd Affiliated Hospital of Zhejiang University Zhejiang University Hangzhou China

## Abstract

*Klebsiella pneumoniae* has emerged as one of the most important pathogens that frequently encounter in community‐acquired or hospital‐acquired infections. Timely epidemiological surveillance could greatly facilitate infection control of *K. pneumoniae* and many deadly pathogens alike. In this study, we evaluated the performance of the IR Biotyper, a Fourier transform infrared (FTIR) spectroscopy system for *K. pneumoniae* isolates typing through (i) optimizing the culture scheme and defining the cutoff value (COV) range and (ii) comparing with commonly used typing tools such as multi‐locus sequence typing (MLST), pulsed‐field gel electrophoresis (PFGE) and whole‐genome sequencing (WGS). We found that a non‐selective and non‐chromogenic medium with 24 ± 2 h incubation gives the best discriminatory power for the IR Biotyper (IRBT). COV evaluation indicated that the IRBT is a robust typing method with good reproducibility. Besides, we observed that the modified H_2_O‐EtOH suspensions preparation method could enhance the quality of the spectrum, especially for those hypermucoviscous strains. For the method comparison study, our data demonstrated that FTIR spectroscopy could accurately cluster *K. pneumoniae* strains. The typing results of the IRBT were almost entirely in concordance with those from PFGE and WGS. Together with the advantages such as low costs and short turnaround time (less than 3h), the IRBT is a promising tool for strain typing that could make real‐time outbreak investigation a reality.

## Graphical abstract

Together with the advantages (low costs and short turnaround time), the IRBT is a promising tool for *K. pneumoniae* typing that could make real‐time outbreak investigation a reality in routine clinical lab.

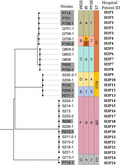

## Introduction

Healthcare‐associated infections (HAIs) post important medical safety issues. It is reported that at least 5–10% of patients admitted to acute care hospitals acquire an infection during hospitalization (Sydnor and Perl, 2011). *Klebsiella pneumoniae*, one of the most frequently isolated gram‐negative bacteria, is often involved in the HAIs. Worse still, it always acquires antibiotic resistance mechanisms allowing itself to tackle the last generation antibiotics (Friedman, et al., 2016). To ensure good hospital hygiene, medical institutions need to implement special hygiene control measures. Early detection and confirmation of pathogens’ cross‐transmissions, especially the multi‐drug resistance ones, are extremely important. Till now, quick observation and approval of pathogen cross‐transmission remain challenging. Although antimicrobial susceptibility testing (AST) can provide some evidence, a robust strain‐level detection method is necessary for epidemiological investigations. Strain typing based on different phenotypic and genotypic principles has been used. Among these, molecular biology methods with relatively high discriminatory power such as multi‐locus sequence typing (MLST) (Maiden, et al., 1998; Enright and Spratt, 1999), and especially the pulsed‐field gel electrophoresis (PFGE), have been widely used for surveillance and outbreak management (Neoh, et al., 2019). Whole‐genome sequencing (WGS), which can provide increased genetic information, has become the new gold standard for investigating suspected outbreaks in hospital settings (Gilchrist, et al., 2015). But the discriminatory power, high cost, laborious and time‐consuming have limited their routine implementation (Sabat, et al., 2013).

Fourier transform infrared (FTIR) spectroscopy is a phenotypic method traditionally used for determining the molecular composition of a wide range of sample types. FTIR can differentiate strains through quantifying the absorption of infrared light by a variety of molecules present in the sample, such as lipids, nucleic acids, proteins, especially the component carbohydrates and lipopolysaccharides from the microbial cell wall (Lasch and Naumann, 2015). In 2017, Bruker launched the IR Biotyper (IRBT), a spectroscopic system for microorganisms typing based on FTIR technology. Unlike previous FTIR‐based typing attempts (Sandt, et al., 2003, Sousa, et al., 2014), the IRBT is an integrated system to provide a turn‐key solution for microorganism typing. Since its introduction, IRBT has been used in the typing of bacterial isolates such as *K. pneumoniae*, *Enterobacter cloacae* and other gram‐negative bacilli, or gram‐positive bacterium like *Streptococcus pneumoniae* and *Staphylococcus aureus* (Johler, et al., 2016, Campos, et al., 2018, Burckhardt, et al., 2019, Martak, et al., 2019). Although these studies demonstrated IRBT’s capability as a typing tool in various degrees, detailed operability and performance investigation in the clinical microbiology laboratory have been lacking.

In the current study, we aim to evaluate the performance of IRBT for *K. pneumoniae* typing of clinical relevance, including optimization of culture scheme, sample preparation method and reference cutoff value (COV) range, which are very important for implementing this new phenotypic typing method into routine use in hospital hygiene management. Besides, we compared its discriminatory power and concordance with other typing methods and assessed its performance in a retrospective outbreak investigation.

## Results

### Culture condition optimization

**Fig. 1:**
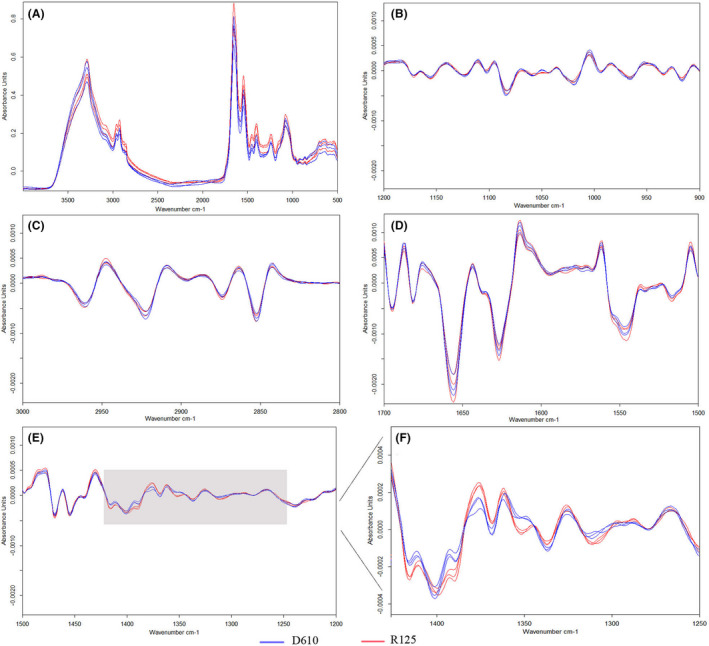
Second derivative FTIR spectra of four biochemical assigned sub‐ranges of D610 and R125 obtained from 24 h incubation time of MH agar plate. A. Typical infrared spectra in the wavenumber 4000–500 cm^−1^ of three replicates of D610 (blue line) and R125 (red line); B. Second‐derivative FTIR spectra in the polysaccharide absorption region (1200–900 cm^−1^); C. Second‐derivative FTIR spectra in the lipids absorption region (3000–2800 cm^−1^); D. Second‐derivative FTIR spectra in the proteins/amides I and II (1700–1500 cm^−1^) regions; E. Second‐derivative FTIR spectra in the mixed region of phospholipids/DNA/RNA (1500–1200 cm^−1^); F. The amplified phospholipids/DNA/RNA region (1425–1250 cm^−1^).

For culture scheme determination, nine *K. pneumoniae* isolates were cultured on four different commonly used mediums [Columbia Sheep Blood Agar plates (BA), China Blue Agar plates (CB), Mueller–Hinton agar plates (MH) and MacConkey Agar plates (MCA)] for 24 ± 2 h and 48 ± 2 h. After cultivation and spectra acquisition, eight projects were used to build the dendrograms for each medium and incubation time (Table 1). The cutoff value obtained automatically ranged from 0.196 (BA 24 h) to 0.439 (MCA 24 h), MCA 24 h result in the maximum COV because of the weak growth of *K. pneumoniae* strains on chromogenic medium and inconsistent spectra quality. All different strains were separated into different IR types using the 24 h incubation time. However, when we postponed the incubation time to 48 h, two strains of R125 and D610, which could be separated before grouped into the same cluster when using BA and CB medium strains (Table 1), this result indicated a more protracted time incubation might change the difference between different *K. pneumoniae* cell wall components and further reduce the discriminatory power of IRBT. Further spectrum analysis of R125 and D610 obtained from 24 hours incubation time of the MH agar plate indicated that no significant difference was observed at polysaccharides (1200‐900 cm^−1^), lipids (3000–2800 cm^−1^) or proteins/amides I and II (1700–1500 cm^−1^) regions. The most pronounced spectral discrepancy was observed at a mixed area of phospholipids/DNA/ RNA (1500–1200 cm^−1^), especially wavenumbers at ˜ 1415, ˜1393, ˜1376, ˜1352 and ˜ 1311 cm^‐1^, showing the least spectrum overlap which represents the most distinction (Fig. 1).

**Table 1:** Comparison of various culture schemes.

Strain NO.	ST	IR typing^a^									
		MH24h	CB24h	BA24h	MCA24h	MH48h	CB48h	BA48h	MCA48h	MH24h + BA24h	MH24h + MH48h
E105	ST101	1	1	1	1	1	1	1	1	1	1
S102	ST280	2	2	2	2	2	2	2	2	2	1
J807	ST11	3	3	3	3	3	3	3	3	3	2
F824	ST23	4	4	4	4	4	4	4	4	4	2^b^
L106	ST152	5	5	5	5	5	5	5	5	5	3
D713	ST111	6	6	6	6	6	6	6	6	6	4
D610	ST1786	7	7	7	7	7	7	7	7	7	5
R125	ST1779	8	8	8	8	8	7	7	8	7	5
E502	ST11	9	9	9	9	9	8	8	9	8	6
Total	–	9	9	9	9	9	8	8	9	8	6
COV	–	0.295	0.275	0.196	0.439	0.249	0.423	0.3	0.268	0.521	0.56

^a^ ST, sequence type; COV, cut‐off value; MH, Mueller‐Hinton agar; CB, China Blue agar; BA, Columbia Sheep Blood agar; MCA, MaConkey agar.

^b^ The replicates of F824 could not be grouped into the same cluster.

All the strains were commendably separated on the same media of twenty‐four‐hours incubation. However, when introducing spectrum from different medium or incubation time, a higher COV or wrong clustering for some strains is observed (Table 1), which indicated that, for *K. pneumoniae,* culture conditions including media and incubation time need to be fixed to ensure a high typing resolution by IRBT. We unified the culture scheme of MH medium, 24 ± 2 h, a non‐selective and non‐chromogenic medium that can get better growth in a shorter time for subsequent experiments.

### Modified sample preparation procedure

**Fig. 2:**
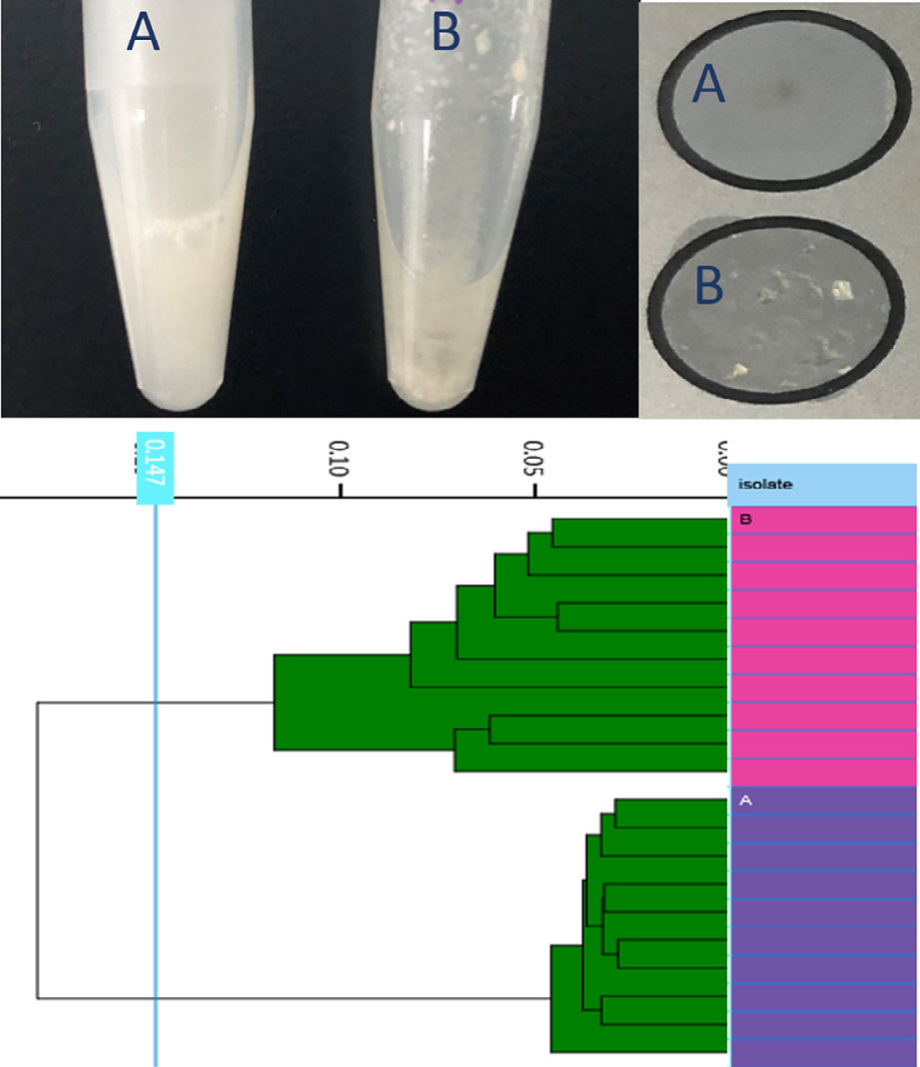
Comparison of the results from the two sample preparation methods. A and B represents samples prepared using modified H_2_O‐EtOH method and official EtOH‐H_2_O method, respectively.

For hypermucoviscous *K. pneumoniae* strains, we found in the preliminary experiments that the official EtOH‐H_2_O method recommended by the manufacturer could be optimized for *K. pneumoniae*; it takes a longer time for suspensions preparation. Sometimes, even after extended vortex, stable and homogeneous suspensions could not be formed, which resulted in a low successful acquisition rate. Compared to the official method, the samples prepared using the modified H_2_O‐EtOH method can get a better solution, a smoother and flat membrane adhered to the plate, and better parallelism of the spectrums acquired from different spots (Fig. 2). After switching the two sample preparation steps, the modified H_2_O‐EtOH method significantly improves the successful acquisition rate (100%) (Data not shown). Based on the improvement in sample suspension and spectral quality, we decided on the modified method in the subsequent experiments for the study.

### Evaluation of Cutoff Value (COV)

**Fig. 3:**
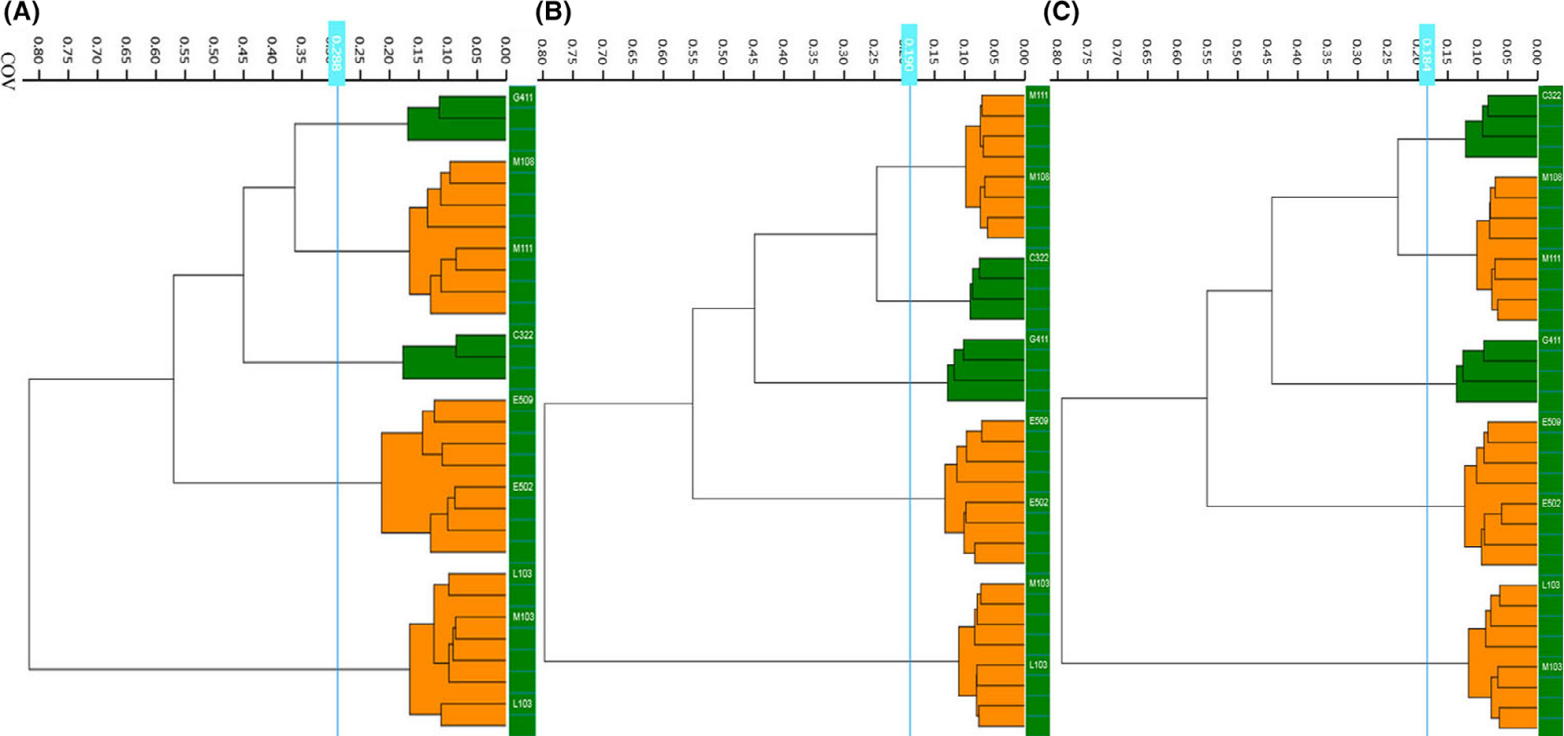
Cut‐off value assessment of eight*K. pneumoniae*isolates. A, B, C represents for strains obtained in three independent days.

Eight *K. pneumoniae* ST11 isolates, which covered five sequence types according to WGS, ranging from a minimum of 11 SNPs to a maximum of 231 SNPs discrepancy, were chosen for COV assessment. The WGS typing results were used as a reference. For each dendrogram from three independent experiments on separate days, IRBT software calculated a cutoff value automatically. The given COVs all gave consistent results as those of WGS, which showed the robustness of IRBT. Considering that the actual cutoff value is set in the ‘middle’ of the distance between the 2 nodes (left/right) form a new cluster and the higher distance value of the 2 current nodes/clusters, the COV range should be 0.288 [0.215, 0.363] (Fig. 3A), 0.190 [0.134, 0.247] (Fig. 3B) and 0.184 [0.135, 0.233] (Fig. 3C). For this close related strain set, 0.221 could be the average COV, and [0.161, 0.281] was selected as the COV range.

### Comparison with other methods

**Fig. 4:**
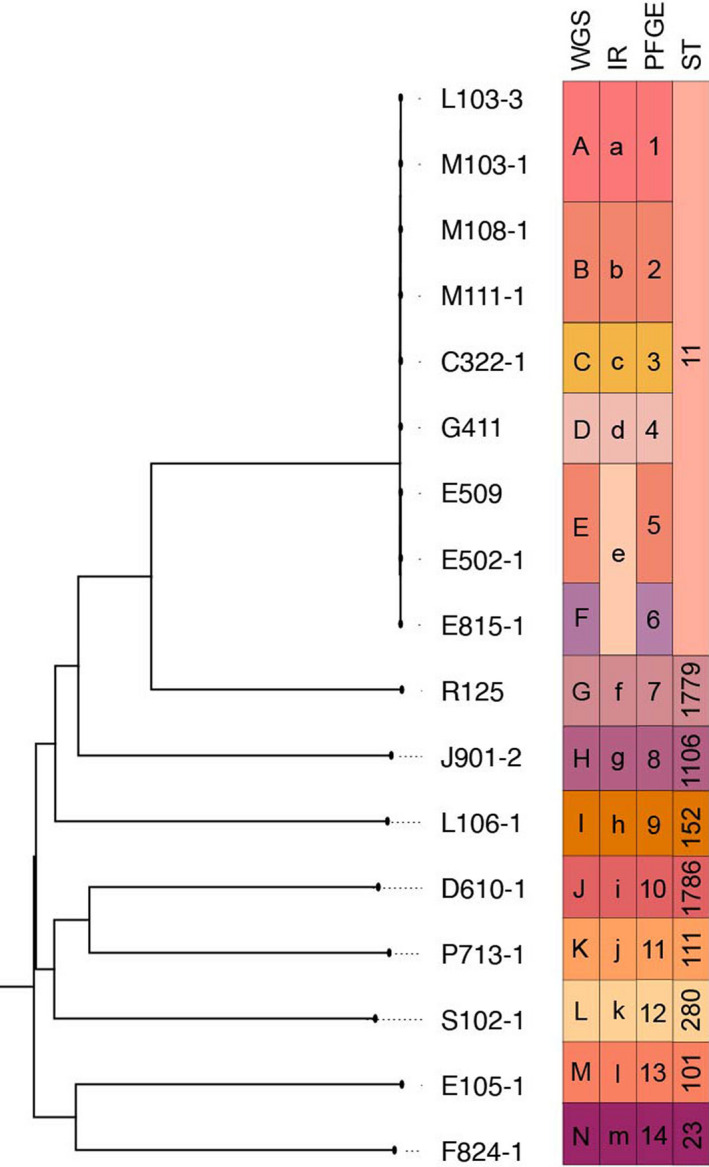
Phylogenetic tree of 17 *K. pneumoniae*isolates. The dendrogram was constructed by SNP‐based core‐genome analysis and was rooted by midpoint without branch transform. The lineages of WGS, IRBT, PFGE, and STs are given for each isolate and denoted by colored stripes.

A comparison of MLST, PFGE, WGS and IRBT is shown in Fig. 4, IRBT can differentiate 17 *K. pneumoniae* isolates from 9 STs into 13 IR types, 10 IR types contained only a single isolate, and nine isolates from ST11 strains were clustered into five different IR types. PFGE and WGS clustering results are entirely consistent with each other; both contain fourteen types and classified ST11 isolates into six classes. Simpson’s index of diversity (SID) was used to determine the discriminatory power of the four typing methods. As shown in Table 2, PFGE and WGS had the highest discriminatory power (0.978) followed by IRBT (0.963), and the lowest SID was from MLST (0.735). The coherence of IRBT to WGS was 92.9%, the only one incongruence strain of IRBT and WGS was E815, which had been clustered into IR type 3 together with E502 and E509. The distance matrix of SNP showed the discrepancy of E815 with E502 and E509 was 36 SNPs and 37 SNPs, respectively, which is relatively a minor difference.

**Table 2:** Comparison of each typing method.

Method	No. of types	Simpson’s ID (95% CI)	Concordance with WGS	Adjusted Rand index			
				MLST	IR	PFGE	WGS
MLST	9	0.735 (0.507–0.963)	52.90%	–	–	–	–
IR	13	0.963 (0.919–1.000)	92.90%	0.192	–	–	–
PFGE	14	0.978 (0.951–1.000)	100%	0.118	0.743	–	–
WGS	14	0.978 (0.951–1.000)	–	0.118	0.743	1	–

The concordance analysis of the four methods was calculated through the adjusted Rand’s index. The highest value of 1.0 was obtained between PFGE and WGS, followed by IRBT typing with these two methods (0.743). The lowest concordance was found between MLST and the other three methods (Table 2).

### Suspected Outbreak investigation

**Fig. 5:**
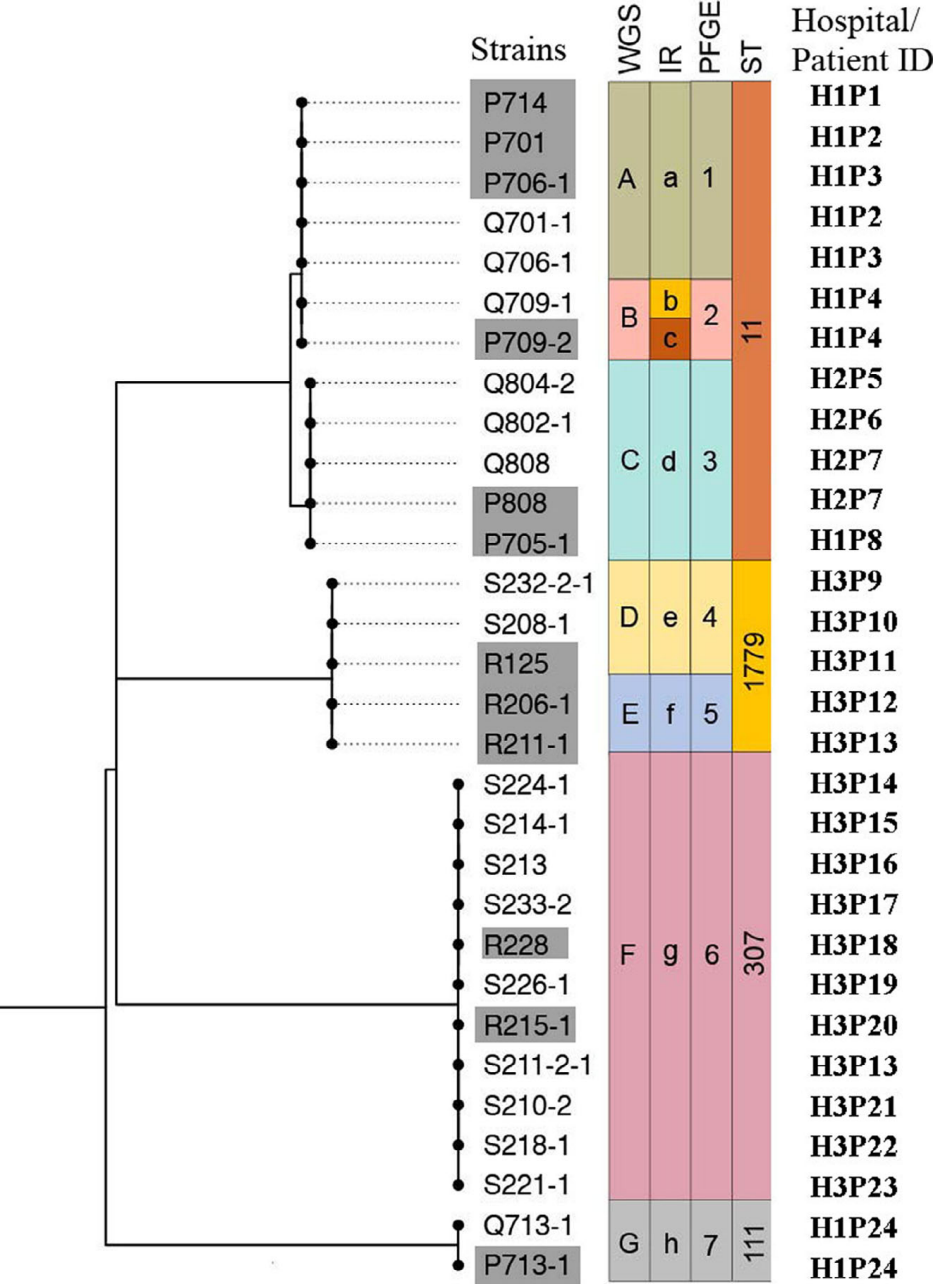
Phylogenetic tree of 30 outbreak investigation*K. pneumoniae*isolates. A dendrogram was obtained by SNP‐based core‐genome analysis and was rooted by midpoint without branch transform. IRBT, Pulsotypes, and STs are given for each isolate and presented as colored stripes. The hospital/patient ID is shown in the text after the corresponding isolate name. Isolates collected from anal swabs were in gray shadow and throat swabs in normal.

A total of 30 *K. pneumoniae* isolates obtained from different departments of three hospitals were analyzed by IRBT, compared with PFGE, WGS and MLST. The definition of a suspected outbreak of healthcare‐acquired infection was determined according to the standards of the National Health Commission of the People’s Republic of China (http://www.emedicine.org.cn/yjdt/905313.htm). Four outbreaks and six outgroups were included in this study. Eight IR types were identified among the 30 isolates, almost the same cluster classification pattern with those of PFGE and WGS (7 types) (Fig. 5), ARI was 0.992 between IR and PFGE/WGS, which indicated a high congruence of the three methods. IRBT can predict all four outbreaks, and no outgroup isolates clustered into the outbreaks.

## Discussion

As a phenotypic analysis method, variation in culture medium, incubation time and other culture parameters, which led to different growth conditions of strains, have a significant impact on the IRBT clustering results. The missed clustering results acquired under different culture schemes demonstrated that it is necessary to fix the culture condition for a robust IRBT typing procedure. In this research, based on the better growth condition, no chromogenic interference and relatively stable results, we select MH medium 24 ± 2 h as the standard culture scheme in our laboratory for IRBT typing of *K. pneumoniae*. We also observe that prolonging the incubation time to 48h lowered the discriminatory power of IRBT on some medium. This conclusion is consistent with the results of Wenning, et al. (2014), which found that the FTIR identification rate significantly decreases when the incubation time was prolonged to 48 or 72 h. It may be because long‐term cultivation changed the difference of the cell wall composition of different strains or led to less reproducible spectra, more in‐depth research work needs to be implemented to verify these hypotheses.

The sample preparation method recommended by the manufacturer may not be optimal for all kinds of microorganisms. For *K. pneumoniae*, it is challenging to get homogeneous suspensions after inactivation in 70% EtOH (maybe due to that 70% EtOH denatures and coagulates bacteria cell protein in contact, and then blocks the homogenization of the suspensions); the modified H_2_O‐EtOH method seems to be a better solution, reduced the suspension preparation time and improved the acquisition success rate significantly. This result also suggests it may be necessary to optimize the sample preparation methods for different microorganisms (especially molds or rough bacteria). Besides, entirely inactivation procedures are also needed when applying to some highly pathogenic microorganisms like Mycobacterium; 75% concentration of ethanol, UV irradiation or other physical inactivated methods might be feasible options.

Before the IRBT, previous studies of FTIR strain typing were commonly based on hardware and third‐party statistics analysis software (Samelis, et al., 2011; Wenning and Scherer, 2013); the integration of hardware and software of IRBT gives a potential turn‐key solution for routine outbreak investigation. Our attempts here to determine the COV for *K. pneumoniae* set precedence for others to deal with different microorganisms. It is hard to fix a specific COV for each microorganism; this is consistent with the conclusion of Martak D (Martak, et al., 2019). But from analysis of 8 closely related ST11 isolates, a COV range [0.161, 0.281] obtained can be used to define whether the isolates tested are closely related (strain level for *K. pneumoniae*); this COV range is similar to the value suggested by the manufacturer [0.2–0.25] for strain‐level typing of *K. pneumoniae*. However, quite different to the results obtained by Rakovitsky, et al. (2020), this may due to the different data exploration algorithm, there are several exploration algorithms preloaded in the IRBT system, we select Euclidean distance and average linkage in this study, and Rakovitsky, et al. (2020) might use the correlation distance and average linkage, as the quite small COV automatic given by their results. Hence, if the COV range needs to be referenced, we recommend adding the data exploration algorithm.

Comparison results demonstrated that the IRBT has higher discriminatory power than MLST (SID 0.963 vs. 0.735), comparable to the previous and present gold standard, PFGE and WGS. In this study, all three technology platforms (IRBT, PFGE and WGS) can consistently distinguish ten different sequence types. It is also worth noticing that there are no contradictory results from IRBT to the other three methods, which means no isolates from the same WGS, PFGE or MLST type clustered into different IRBT types. IRBT, PFGE and WGS can further discriminate ST11, which is the most predominant sequence type in China, into different types. The only one discordance between the IRBT and the WGS was E815. It has only 36 and 37 SNPs discrepancy to the other two isolates, which were clustered together by the IRBT; the high degree of similarity may explain the clustering result of IRBT. However, there is no evidence that ˜ 35 SNPs are the limit of IRBT’s discriminatory power. No definite correlation between IRBT types and SNPs difference was observed so far. For example, D610 and R125, which are different sequence types with 27 006 SNPs discrepancy, always showed minimal distance (˜ 0.25) on the IRBT dendrogram, even smaller than most of the distance between two ST11 types. According to the manufacturer’s instrument default setting, the observed spectra range of IRBT focuses on poly‐saccharide regions (1200–900 cm^−1^), which showed conspicuous discrepancy among different microorganisms most of the time. However, it could not be ruled out the possibility that the genotype difference will be reflected in other regions, like fatty acids, proteins or other combined areas. As an example, the second derivative FTIR spectra of four biochemical assigned sub‐ranges of D610 and R125 are shown in Fig. 1. Therefore, further adjusting the spectra range could be a solution to improve the discriminatory power of IRBT.

A retrospective outbreak study of *K. pneumoniae* was conducted to evaluate the effectiveness of the IRBT in the routine infection control situation. We found that the IRBT can correctly cluster isolates by outbreaks (AR = 0.992 with PFGE/WGS). What the most impressive is the IRBT could report the results in less than 3h at a fraction of the cost of PFGE, MLST or WGS. (The turnaround time for PFGE, MLST and WGS is at least 3 days, 2–3 days and 30 days, respectively.) Moreover, PFGE is much more laborious, and even though the cost of WGS would come further down and becomes possible to perform the sequencing and analysis in < 24 h in the future, it is still hard to imagine it could become a routine laboratory tool at any time soon. All these showed the IRBT has the potential to be implemented in a clinical laboratory routine for outbreak investigation and fast and effective infection control.

In summary, this is the first comprehensive suitability study of IRBT. Our research shows that FTIR is a powerful tool for the typing of *K. pneumoniae*. Unlike those current molecular typing methods that only allow a retrospective analysis of outbreak, the advantages of the IRBT include ease of use and fast turnaround time with relatively high discriminatory power. Together with relatively low running costs, the IRBT seems to be a good fit for real‐time surveillance of outbreaks in the clinical lab.

## Experimental procedures

### Bacterial isolates

A total of 47 carbapenem‐resistant *K. pneumonia* strains collected from January 2018 to December 2018 in a Chinese clinical microbiology laboratory were used in the current study. The strains were recovered from the routine microbiological throat or anal swab screening samples taken from intensive care units. Specifically, nine isolates with eight different sequence types (STs) were obtained for the screening of the optimal culture condition, and eight strains with the same ST (ST11), which were differentiated by WGS into five different types, were chosen for COV evaluation. For the method comparison investigation, 17 strains from 9 STs were evaluated. The other 30 isolates recovered from Hunan province were used for retrospectively suspected outbreak analysis compared with the other methods. Strains were recovered on Columbia sheep blood agar plates (Autobio Diagnostics, Zhengzhou, China) for 24 ± 2 h at 35 °C for MLST, PFGE and WGS analysis. For FTIR analysis, strains were grown on the corresponding plates at 35 for 24 ± 2 or 48 ± 2 h. All the above isolates were confirmed by matrix‐assisted laser desorption/ionization time‐of‐flight mass spectrometry (MALDI Biotyper; Bruker Daltonik GmbH, Bremen, Germany) at species level when they were used for MLST, PFGE, WGS and FTIR analysis.

### PFGE

All the 47 *K*. *pneumoniae* isolates were subjected to *XbaI*‐digested PFGE typing, as previously described (Hu, et al., 2012). *Salmonella enterica* serotype Braenderup H9812 was used as a size marker. A dendrogram was generated from the homology matrix with a coefficient of 0.5% using the unweighted pair group method using arithmetic averages (UPGMA) to describe the relationships among PFGE profiles. Isolates were considered the same PFGE group if their Dice similarity index was ≥ 80% (Maule, 1998).

### WGS and MLST analysis

Genomic DNAs were extracted from overnight cultures using the Wizard Genomic DNA Purification Kit (Promega) following the manufacturer’s instructions and were subjected to whole‐genome sequencing using 150 bp pair‐end strategies with the Illumina HiSeq X10 platform. Raw reads were trimmed and assembled to contigs using SPAdes v3.11.1 (Bankevich, et al., 2012). Genome sequences were annotated with the RAST tool (Overbeek, et al., 2014). Multilocus sequence types were determined by using the SRST2 toolkit version 0.2.0. (Inouye, et al., 2014). The evolutionary relationships were analyzed by parsnp software based on the core genomes, and the trees were visualized with FigTree software (Version 1.4.3) and Adobe Illustrator software (Version 22.1). Single‐nucleotide polymorphisms (SNPs) were called by the gingr software of Harvestsuite. The Harvest suite is open source and freely available from:http://github.com/marbl/harvest. Isolates, with an SNP discrepancy of ≤ 18, were considered orthologous (Schurch, et al., 2018).

### FTIR spectroscopy analysis

Firstly, for culture scheme determination, nine strains were cultured under 35 without the addition of CO_2_ on four different mediums [Columbia Sheep Blood Agar plates (BA), China Blue Agar plates (CB), Mueller–Hinton agar plates (MH) and MacConkey Agar plates (MCA)] for 24 ± 2 h, and 48 ± 2 h. For the other analysis, all the isolates were grown at 35 without the addition of CO_2_ on the MH medium for 24 ± 2 h.

Secondly, two sample preparation methods were applied for FTIR in the current study. After incubation, according to the manufacturer’s instruction (EtOH‐H_2_O method), take a loopful of bacteria into 100 μl of 70% (vol/vol) ethanol to 1.5‐ml vials containing sterile metal rods, vortex, and then, add 100 μl sterile H_2_O into the suspension, vortex 30 s. For the modified procedure, switch the sequence of the two steps (H_2_O‐EtOH), first, take a loopful of bacteria growth into 100 μl sterile H_2_O, and then, add 100 μl 70% (vol/vol) ethanol after vortex. After getting homogeneous suspensions, take 15 μl of the suspension and spot onto the IRBT silicon sample plate (Bruker Daltonik, Bremen, Germany), and dry under 37°C until a dry film is formed on the plate; three or four replicates were prepared for each sample.

Thirdly, for COV evaluation, twelve consistent spectra of each isolate were acquired corresponding to three biological replicates (obtained in three independent days and fresh cultures) and four technical replicates (obtained on the same day from the same agar plate).

Insert the dried sample plate into the IRBT, edit a new run, and start acquisition using the IRBT Client software (version 2.0) with the default analysis settings. Generally, spectra (4000–500 cm^‐1^) of isolates and background were acquired. For each spectrum, 64 scans were collected in single‐beam mode with 4 cm^−1^ resolution at a rate of 3 scans/s. And the spectra range of 1200–900 cm^‐1^ was automatically processed, normalized and second derivative by OPUS7.5 software (Bruker Optics GmbH). Data that did not meet the default quality criteria (0.4 < absorption < 2, signal/noise > 40, signal/water > 20, fringes [×10^‐6^]<100) were excluded in the further analysis. All qualified data were analyzed using offline IRBT Client by building dendrograms using Euclidian distance and average linkage clustering method following the manufacture’s suggestion.

### Calculation of clustering concordance

We use the online tool (www.comparingpartitions.info) to assess the quantitative data of discriminatory power and concordance of the typing methods. The discriminatory power of each typing method was evaluated with Simpson’s index of diversity (SID), calculating the probability that two unrelated isolates from the test strain set will be clustered into different typing groups. Adjusted Rand index (ARI) with 95% confidence intervals was used to evaluate the concordance of IRBT typing results with other methods (Carrico, et al., 2006).

## Conflict of interest

None declared.

## Ethical approval

Bacterial isolates used in this study were previously approved by the Ethics Committee of Second Affiliated Hospital of Zhejiang University, School of Medicine (Number: 2019‐074). All the specimens were anonymized, and patients were not physically involved in this study. No consent was needed for this study.
